# Influence of New Technologies on Post-Stroke Rehabilitation: A Comparison of Armeo Spring to the Kinect System

**DOI:** 10.3390/medicina55040098

**Published:** 2019-04-09

**Authors:** Aušra Adomavičienė, Kristina Daunoravičienė, Raimondas Kubilius, Lina Varžaitytė, Juozas Raistenskis

**Affiliations:** 1 Department of Rehabilitation, Physical and Sports Medicine, Faculty of Medicine, Vilnius University, Santariskiu g.2, LT-08661 Vilnius, Lithuania; juozas.raistenskis@santa.lt; 2 Department of Biomechanical Engineering, Vilnius Gediminas Technical University, J. Basanavičiaus g. 28, 03224 Vilnius, Lithuania; kristina.daunoraviciene@vgtu.lt; 3 Rehabilitation Department, Lithuanian University of Health Sciences, Eiveniu g. 2, LT-50161 Kaunas, Lithuania; raimondas.kubilius@kaunoklinikos.lt (R.K.); lina.varzaityte@gmail.com (L.V.)

**Keywords:** stroke rehabilitation, hand motor function, cognitive recovery, Kinect, Armeo

## Abstract

*Background*: New technologies to improve post-stroke rehabilitation outcomes are of great interest and have a positive impact on functional, motor, and cognitive recovery. Identifying the most effective rehabilitation intervention is a recognized priority for stroke research and provides an opportunity to achieve a more desirable effect. *Objective*: The objective is to verify the effect of new technologies on motor outcomes of the upper limbs, functional state, and cognitive functions in post-stroke rehabilitation. *Methods*: Forty two post-stroke patients (8.69 ± 4.27 weeks after stroke onset) were involved in the experimental study during inpatient rehabilitation. Patients were randomly divided into two groups: conventional programs were combined with the Armeo Spring robot-assisted trainer (Armeo group; *n* = 17) and the Kinect-based system (Kinect group; *n* = 25). The duration of sessions with the new technological devices was 45 min/day (10 sessions in total). Functional recovery was compared among groups using the Functional Independence Measure (FIM), and upper limbs’ motor function recovery was compared using the Fugl–Meyer Assessment Upper Extremity (FMA-UE), Modified Ashworth Scale (MAS), Hand grip strength (dynamometry), Hand Tapping test (HTT), Box and Block Test (BBT), and kinematic measures (active Range Of Motion (ROM)), while cognitive functions were assessed by the MMSE (Mini-Mental State Examination), ACE-R (Addenbrooke’s Cognitive Examination-Revised), and HAD (Hospital Anxiety and Depression Scale) scores. *Results*: Functional independence did not show meaningful differences in scores between technologies (*p* > 0.05), though abilities of self-care were significantly higher after Kinect-based training (*p* < 0.05). The upper limbs’ kinematics demonstrated higher functional recovery after robot training: decreased muscle tone, improved shoulder and elbow ROMs, hand dexterity, and grip strength (*p* < 0.05). Besides, virtual reality games involve more arm rotation and performing wider movements. Both new technologies caused an increase in overall global cognitive changes, but visual constructive abilities (attention, memory, visuospatial abilities, and complex commands) were statistically higher after robotic therapy. Furthermore, decreased anxiety level was observed after virtual reality therapy (*p* < 0.05). *Conclusions*: Our study displays that even a short-term, two-week training program with new technologies had a positive effect and significantly recovered post-strokes functional level in self-care, upper limb motor ability (dexterity and movements, grip strength, kinematic data), visual constructive abilities (attention, memory, visuospatial abilities, and complex commands) and decreased anxiety level.

## 1. Introduction

Insufficient motor control compromises the ability of Stroke Patients (SP) to perform activities of daily living and will likely have a negative impact on the quality of life. Improving Upper Limb (UL) function is an important part of post-stroke rehabilitation in order to reduce disability [1]. Recovery in the context of motor ability may refer to the return of pre-stroke muscle activation patterns or to compensation involving the appearance of alternative muscle activation patterns that attempt to compensate for the motor function deficit [2]. The past decades have seen rapid development of a wide variety of assistive technologies that can be used in UL rehabilitation. These include electromyographic biofeedback, virtual reality, electromechanical and robotic devices, electrical stimulation, transcranial magnetic stimulation, direct current stimulation, and orthoses [3]. Currently, two effective technologies that provide external feedback to SP during training, improve the retention of learned skills, and may be able to enhance the motor recovery are discussed [4].

Virtual Reality (VR): The Microsoft TM Kinect-based system provides feedback on movement execution and/or goal attainment [5]. Incorporating therapy exercises into virtual games can make therapy more enjoyable and more realistic, such that task-based exercises have increased applicability in the clinical environment [6,7], increasing motivation and therefore adherence, which are useful for navigating this virtual environment; this has been identified as the most feasible for future implementation [7].

Electromechanical and robotic devices can move passive UL along more secure movement trajectories and provide either assistance or resistance to movement of a single joint or control of inter-segmental coordination. Recent technological advances have the ability to control multiple joints accurately at the same time, enabling them to produce more realistic task-based exercises for SP [8]. Compared to manual therapy, robots have the potential to provide intensive rehabilitation consistently for a longer duration [9]. Recovery of sensorimotor function after CNS damage is based on the exploitation of neuroplasticity, with a focus on the rehabilitation of movements needed for self-independence. This requires physiological limb muscle activation, which can be achieved through functional UL movement exercises and activation of the appropriate peripheral receptors [10]. The Armeo Spring robot-assisted trainer device may improve UL motor function recovery as predicted by reshaping of cortical and transcallosal plasticity, according to the baseline cortical excitability [11]. Knowledge of the potential brain plasticity reservoir after brain damage constitutes a prerequisite for an optimal rehabilitation strategy [12,13]. There is evidence that robot training for the hand is superior; during post-stroke rehabilitation, hand training is likely to be the most useful [8,13].

Previous studies have shown that the use of systems based on VR environments, motion sensors, and robotics can improve motor function. Currently, no high-quality evidence can be found for any interventions that are currently used as part of routine practice, and evidence is insufficient to enable comparison of the relative effectiveness of interventions [14,15,16].

The objectives of the study are to clarify in which area of functional UL recovery these new technologies are more suitable and effective and how much these interventions affect functional state and cognitive functions.

We raise the hypothesis that a robot-assisted device and virtual reality both have a positive effect on functional independence recovery in stroke-affected patients; however, having a different influence on UL motor function and cognitive changes. We assume that the robot-assisted device is more efficient and more accurately allows selecting tasks for developing specific motor function (range of motion, strength or dexterity of the affected arm), while Kinect-based games provide more free movements that are less suitable for specific motor function development and may be more targeted for cognitive functions.

## 2. Materials and Methods

### 2.1. Selection and Description of Participants

**Figure 1:**
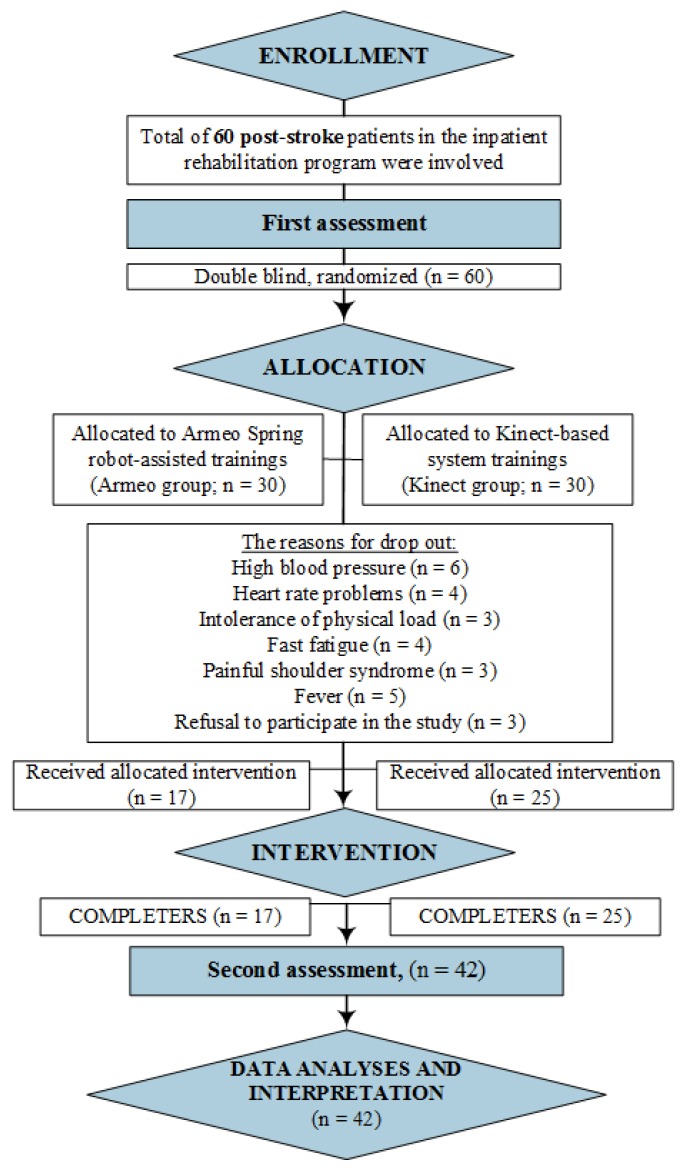
Study flowchart for patients’ selection, assessment, and analysis.

According to very strict inclusion criteria and a small number of patients undergoing post-stroke rehabilitation in our inpatient unit (<70–80 patients/year), a total of 60 post-stroke patients, i.e., 30 patients in each group, were involved in a randomized prospective clinical trial. However, some of the patients did not complete the research due to worsening health: fever, high blood pressure, heart rate problems, and intolerance of physical load. A total of 42 patients completed the study, and their results were analyzed. The main steps of our study are presented in the flowchart in Figure 1.

The following were the inclusion criteria: (1) ischemic or hemorrhagic stroke (confirmed by neuroimaging tests), (2) 60–74 years old (according to WHO’s definition of elderly people), (3) stroke-affected arm paresis, (4) disturbed deep and superficial sensations, and (5) MMSE score >21 points (no cognitive impairment 24–30 or mild impairment 18–23 points). Exclusion criteria were the following: (1) stroke-affected arm paralysis, (2) MMSE score <21 points (severe cognitive impairment = 0–17 points), (3) aphasia, (4) painful shoulder syndrome, and (5) hypertonic stroke-affected arm (≥2 according to the Modified Ashworth Scale).

Patients were randomly (i.e., by chance) assigned to either a treatment based on a combination of a conventional rehabilitation program with the Armeo Spring robot-assisted trainings (Armeo group (AG); *n* = 17) and with the virtual reality Kinect-based system trainings (Kinect group (KG); *n* = 25) to measure and compare the effect and value of a treatment against different technologies. All patients gave their written informed consent. The study was approved by the Vilnius Regional Biomedical Research Ethics Committee, No. 158200-17-912-422.

The conventional post-stroke rehabilitation program lasted 4–3 h daily, 5 days/week (physical therapy, occupational therapy, neuropsychological training, speech therapy, etc.), and the duration of sessions with new technological devices (Kinect or Armeo robot) was 45 min/day (10 sessions in total). All training sessions consisted of a sequence of motor tasks followed by a short resting phase. The patients were asked to perform a large variety of tasks and the exercise program selected individually for each patient. All patients completed 45 min per day for a total of 10 intensive therapy sessions. The trainings were supervised by an occupational therapist who modified the exercise program according to each patient’s progress. At the start of each training session, the clinician examined arm impairment to investigate motor function recovery and pain or other complications. All patients sat on a chair or wheelchair fitted with seat belts to limit torso movements and prevent falling. Patients were instructed to contribute actively to the exercise according to the goal movements.

### 2.2. Technical Information

The main outcome measures used for evaluation of UL recovery after stroke were the following: the Fugl–Meyer Assessment Upper Extremity (FMA-UE, 0 = lowest score; 66 = highest score) [8,13,17]; the shoulder, elbow, and wrist flexion tones were assessed by using the Modified Ashworth Scale (MAS, range 0–4 points) [8]; the gross manual dexterity were assessed by Box and Block Test (BBT, count of cubes during 60 s) [8]; the Hand Tapping Score Test (HTS, score(s) of 25 arm movements) [14]; and to obtain kinematic parameters of requested movements, active Range Of Motion (ROM) of the shoulder, elbow, and wrist was measured [9], and the affected hand grip strength was measured with a hand-grip dynamometer (average of grip strength in kg). The Modified Functional Independence Measure (FIM, 6-item self-care, max 42 point) [15] was used for evaluation of the degree of independence and assistance needed in activities of daily living; to measure cognitive impairment, the Mini-Mental State Examination (MMSE, range 21 ≥ 30 point) [16] and Addenbrooke’s Cognitive Examination-Revised (ACE-R, max 100 points; a higher score reflects a better outcome) were used [18], and the psycho-emotional state was assessed by the Hospital Anxiety and Depression Scale (HAD, range 0–21 points; a higher score reflects higher anxiety and depression level) [19]. The following assessments were extracted at the beginning (pre-outcomes) and at the end (post-outcomes) of the therapy.

In order to investigate the effectiveness of VR-based rehabilitation on UL functional recovery after stroke, the Microsoft Kinect for Windows Software Development Kit (SDK) 2.0 was used: a sensor is equipped with a webcam, a depth camera, and a microphone array designed to facilitate a natural user interface [13]. These trainings consisted of exercise games (the term “exergames” is also commonly used) in a variety of areas. Exergames aim to combine natural human movements and the entertainment of video games to promote patient exercise, according to each patient’s progress. For robot-assisted treatment, the Armeo Spring (Hocoma AG, Volketswil, Switzerland) as used to train UL motor function. During the first session, the device was adjusted for the patient’s arm size and the required angle of suspension (45° shoulder flexion and 25° elbow flexion, approximately). After the UL had been fitted to the system, the working space was measured, and the exercises the patient was able to perform were selected from a large variety of tasks. At the end of robot-therapy sessions, patients were asked to perform specific evaluation exercises making use of virtual reality software, then the working space and the angle of suspension were recalibrated. After a treatment session, the following outcome measures were assessed: Active Range Of Motion (A-ROM) [9], active motion in 3D space (A MOVE), and movement quality in percent. For clinical assessment of affected arm capability, goniometers were used [12].

### 2.3. Statistics

A statistical power analysis was performed in order to estimate the sample size. It was determined that a total of 70 post-stroke individuals would be needed to detect a difference between groups, with a one-tailed α of 0.05 and a (1-β) of 0.95. The G-power software package (Version 3.1.2.9; Franz Faul, University of Kiel, Kiel, Germany) was used to calculate the required sample size of subjects with a significance level of 0.05 and a power of 0.95. Frequencies were calculated for categorical variables. For continuous variables, means ± standard deviations (SD) were obtained. The Shapiro–Wilk normality test (*p* < 0.05) was used to verify data normality. Normally-distributed data were compared by means of a parametric statistical method, i.e., with one-way ANOVA (*p* < 0.05). Non-normally-distributed (*p* < 0.01) and normally-distributed data were compared by means of a non-parametric statistical method, i.e., with the Kruskal–Wallis test (*p* < 0.05).

## 3. Results

Our study included 42 patients (mean age ± standard deviation of 64.6 ± 4.2 years old) with hemiparesis secondary to ischemic (*n* = 29) or hemorrhagic stroke (*n* = 13), who participated consecutively in a specialized rehabilitation unit for a post-stroke time of 8.69 ± 4.27 weeks. In both groups, male participants and the right hand being affected dominated (Table 1).

All the results were obtained by comparing the impact of new technologies on UL recovery. In particular, all data were divided into three important parts: functional independence in daily life activities, UL motor abilities, and cognitive functions. The level of functional independence increased statistically in both groups, but after Kinect-based rehabilitation, SP demonstrated a higher level of independence in self-care activities (*p* < 0.05) than after robot training (Table 2).

A significantly different effect of new technologies for the recovery of Upper Limb (UL) motor capability was not found (Table 3) According to the Fugl–Meyer Assessment Upper Extremity (FMA-UE) results, we can state that result of the mobility of the hemiparetic arm, including reflexes, the appearance of synergies, and each of the isolated movements of the upper limb, including grip, was improved in both groups (*p* > 0.05). However, the flexion muscle tone in the elbow and wrist was increased more in KG (Modified Ashworth Scale (MAS) score), which could limit the arm dexterity; therefore, the results of repetitive movements for 60 s (tapping test) and the box and block score of the affected arm increased more after robot trainings (*p* > 0.05). Hand grip strength recovered significantly more in AG, because of visibly lower muscle tone in the wrist and elbow that allowed patients to carry out these actions more easily.

SP after Armeo training had better kinematic responses in the active range of motion in shoulder extension and flexion. Meaningful statistical differences were found only in shoulder adduction and abduction (*p* < 0.05). However, rotational ROMs of the shoulder appeared improved after Kinect-based training, while all elbow ROMs increased in the AG group. A significant increase in elbow supination (*p* < 0.05) occurred after Armeo robot training. Wrist movements did not show any significant difference between Armeo- and Kinect-based trainings (Table 4).

The changes in SP cognitive functions were reflected in the MMSE, ACE-R and HAD test scores. At the beginning of the sessions, all participants’ MMSE test scores were >21 points: in AG, the mean MMSE score was 24.06 ± 2.44 points and in KG, 23.2 ± 2.27 points (*p* > 0.05). Total cognitive changes due to MMSE were statistically higher in AG (*p* < 0.05) (Table 5).

**Figure 2:**
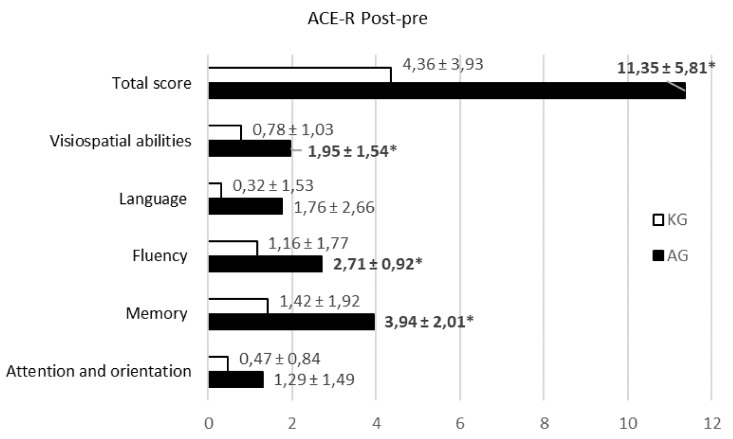
The Addenbrooke’s Cognitive Examination-Revised (ACE–R score) (post-pre) distribution for different abilities in the groups (*n* = 36). Values are represented as the mean ± SD. The Kruskal–Wallis test was used for comparison.

Nevertheless, further investigation of ACE-R scores for different abilities indicated significant differences in memory, fluency, and visuospatial abilities (*p* < 0.05) (Figure 2).

HAD score displayed the following: depression remained unchanged for both groups (*p* > 0.05), and anxiety was decreased by significantly more in KG (*p* < 0.05) (Table 6).

## 4. Discussion

It is not easy to measure the effectiveness of post-stroke rehabilitation treatment to restore lost UL functions [4]. The impact of new technologies on restoring functional state is not well defined. Recent scientific sources provide different criteria and sizes for the effectiveness of these technologies [7]. However, previous research has confirmed that new modern interventions have only a positive impact, be it large or small, on stroke rehabilitation [11,20]. We compared the influence of two new technologies, Armeo vs. Kinect, on the recovery of UL functions in post-stroke rehabilitation. Finally, the study showed clinical improvement in subjects who participated.

A detailed analysis of obtained outcomes was performed, and all results were divided into three main parts: functional independence, motor abilities of UL, and cognitive functions. Notably, the period from pre- to post-treatment was relatively short (two weeks (10 sessions)), and even so, statistically-significant results reflecting the effectiveness of new technology were obtained. Interestingly, the FIM test showed significantly better results in the KG group (*p* < 0.05). This result suggests that VR intervention helps to recover the UL motor function. Our findings are consistent with a study by Webster and Celik, who displayed that rehabilitation was promoted by the enhanced feedback in a virtual environment and that kinematic analysis of movements showed significant improvement on the functional level, which indicated motor recovery in post-stroke patients [21].

UL kinematics demonstrated increased functional recovery after robot training (AG group): decreased muscle tone, improved shoulder extension and flexion, increased elbow ROMs, a meaningful increase in elbow supination, and greater recovery of hand dexterity and grip strength (*p* < 0.05). Our findings only confirmed the fact that robotic devices can improve UL functions by moving passive limbs along more secure movement trajectories and provide either assistance or resistance to movement of a single joint or control of inter-segmental coordination. Recent technological advances in robot training have the ability to control multiple joints accurately and have a positive impact on UL recovery [10,12,22]. Besides, improved rotational ROMs of the shoulder were observed in the KG group. This can be attributed to the fact that Kinect games involve more rotation than the Armeo Spring robot allows. Furthermore, we must state that a robot-assistive device provides affected UL weight support and provides an opportunity to make the most accurate tasks during trainings, while Kinect does not provide UL weight support, and the movements of the affected arm are less accurate and wider. In most cases, as other works report, UL recovery ability depends on specific and precise training tasks, duration, exercising, and intensive repetition of specific movements, while the task’s completion time seems to improve regardless of the training method [5,10]. Furthermore, our research indicated a tendency for muscle tone increase in the elbow and wrist joints for SP who underwent VR interventions. This likely demonstrates the effectiveness of new technologies, but in truth, increased muscle tone limits UL functional recovery. Without a doubt, free hand movements during gaming interfere with the precision of UL motion, unlike in Armeo training. Furthermore, some factors (unstable body position, fall risk, compensated movements, and limited range of motions) during Kinect-based training could affect motor recovery [21].

Our study demonstrated good participant satisfaction and a positive impact on the psycho-emotional state. It is well known that cognitive functions are very important for successful and effective post-stroke rehabilitation. We observed that, during training, variations in cognitive function due to ACE-R and MMSE testing results showed increased visual constructive abilities in SP. Overall global cognitive changes due to MMSE were statistically higher in AG. These testing results were more sensitive to detecting changes in attention and especially in complex commands (executing a command, drawing or copying two pentagons) (*p* < 0.05). According to the ACE-R testing results, greater improvements in memory, fluency, and visuospatial abilities were observed in the AG group (*p* < 0.05). Memory improvements were consistent with a study by Gamito and colleagues [23], which compared a VR-based intervention with conventional rehabilitation and observed that the application of virtual reality-based serious games in cognitive rehabilitation is an effective cognitive training tool for developing SP attention and memory tasks consisting of daily life activities. The visuospatial improvements were consistent with a study by Kim and colleagues [24], which compared a VR-based intervention with a computer-based intervention. Patients interacted with displayed images, moved and manipulated virtual objects, and performed other actions in a way that attempted to “immerse” them in the simulated environment, thereby engendering a feeling of presence in the virtual world, and encouraging reflection, copying, repetition, and prediction [21,25].

Negative psychological outcomes occur frequently after stroke; one of the most common is anxiety disorders and anxiety symptoms, which often have an impact on rehabilitation outcomes. Although stroke rehabilitation is effective at decreasing anxiety symptoms, its effects in tandem with depression remain unclear [26]. Such research confirms our study results (HAD scale): SP felt a greater positive effect of training and significantly decreased anxiety (*p* < 0.05). This shows that participation in even a short, two-week period of rehabilitation can improve the outcome in terms of the psycho-emotional state and reduce anxiety symptoms.

We acknowledge the limitations of our study. Despite the positive impact of new technologies, some limitations of our study must be considered when interpreting the results. Concerning the sample, 42 participants can be considered a small number, though it is comparable with previous similar interventions [23]. Moreover, the study was performed shortly after the stroke had occurred, and the training was only performed for a small number of sessions (10 sessions, 45 min/day). Furthermore, we observed that a 60-min duration of training with new technologies was too long for patients: after 30 min, they became tired, could hardly gather their attention, made long pauses, and did not perform the task correctly. Considering these indicators, the optimal training session duration we recommend is 30 min. Furthermore, we can state that effective results in post-stroke rehabilitation with new technologies depend on training duration and intensity, as well as the patient distribution according to sex, different affected arm (R/L), and stroke type (ischemic/hemorrhagic) with different recovery curves and the psycho-emotional state.

Our hypothesis was confirmed, that trainings with new technologies showed significantly improved functional level of post-stroke patients. However, the effects of trainings on upper limbs’ motor function recovery demonstrate that Armeo robot-assisted devices present the same tracking capabilities as the Kinect-based system, while being individually modified for each patient and accurately overcoming limitations. Thus, the potential efficacy of Kinect and Armeo therapy in the rehabilitation of post-stroke survivors needs to be investigated in greater depth.

## 5. Conclusions

Recently, new techniques based on robotic-assistive devices or VR have proven increasingly beneficial. Our research has shown that even a short-term, two-week training program with new technologies had a positive effect and significantly recovered SP functional level in self-care, UL motor ability (dexterity and movements, grip strength, kinematic data), visual constructive abilities (attention, memory, visuo-spatial abilities, and complex commands), and decreased anxiety level. Obviously, in order to achieve the recovery of a specific impairment, it is very important to select accurately the most appropriate method for effective recovery. These findings suggest the need to explore and carefully plan strategies to stimulate positive improvement within rehabilitation.

## Figures and Tables

**Table 1:** Clinical and demographic characteristics of Armeo group (AG) and Kinect group (KG) (*n* = 42).

Variables	AG (*n* = 17)	KG (*n* = 25)	*p*-Value
Age (years)	66 (60.5–70)	62 (61–69)	>0.05 ^K^
Gender (M/W)	11/6	17/8	>0.05
Affected arm (R/L)	11/6	14/11	>0.05
Stroke onset (weeks)	7 (6–11.5)	7 (5–12) 16/9	>0.05 ^K^
Stroke type (I/H)	13/4		>0.05

Values are represented as the median (interquartile range). ^K^ Kruskal–Wallis test. Stroke onset is the number of weeks before the beginning of a treatment session. L, Left; M, Men; NA, Not Applicable; R, Right; W, Women; H, Hemorrhagic; I, Ischemic.

**Table 2:** Functional independence testing results (*n* = 42). FIM, Functional Independence Measure.

Variables	AG (*n* = 17)	KG (*n* = 25)	*p*-Value
**FIM Score**			
Pre	83.00 ± 14.49	71.68 ± 19.89	>0.05 ^A^
Post	98.29 ± 12.86	97.16 ± 10.02	>0.05 ^A^
Post-pre difference	15.29 ± 4.52	25.48 ± 18.48	<0.05 ^A^*
**Modified FIM Score (Self-Care)**			
Pre	24.41 ± 5.18	21.40 ± 6.60	>0.05 ^A^
Post	31.94 ± 4.39	32.24 ± 3.18	>0.05 ^A^
Post-pre difference	7.53 ± 2.50	10.84 ± 6.47	<0.05 ^A^*

Values are represented as the mean ± SD. ^A^ ANOVA test. Post-pre, the effect of treatment or changes between post-treatment and baseline.

**Table 3:** UL motor function results (*n* = 42).

Variables	AG (*n* = 17)	KG (*n* = 25)	*p*-Value
**FMA-UE (Fugl–Meyer Assessment Upper Extremity**			
Pre	39 (18–47)		>0.05 ^K^
Post	54 (26–59)	33 (23–41) 46 (42–55)	>0.05 ^K^
Post-pre difference	13 (12–15.5)	10 (7.5–22)	>0.05 ^K^
**Modified Ashworth Scale (MAS Score, 0/1/1+ points)**			
**Shoulder**	(*n* = 42)	(*n* = 42)	
Pre	14/2/1	21/3/1	NA
Post	13/4/0	20/3/0	NA
**Elbow**			
Pre	12/1/2	15/5/2	NA
Post	12/2/2	14/6/3	NA
**Wrist**			
Pre	12/2/3	15/4/3	NA
Post	11/1/2	13/4/4	NA
**Hand dynamometry**	20.29 ± 13.3/26.11 ± 18.5	21.48 ± 14.5/23.56 ± 17.2	
(R/L; kg)	24.29 ± 13.0/28.70 ± 18.1	23.72 ± 13.2/25.60 ± 17.2	
Post-pre difference	4.00 ± 0.33/2.59 ± 0.5	2.24 ± 1.2/2.04 ± 0.6	<0.05 ^A^*/>0.05 ^A^
**Hand tapping score**	20.29 ± 13.3/26.11 ± 18.5	21.48 ± 14.5/23.56 ± 17.2	
(L/R, seconds)	24.29 ± 13.0/28.70 ± 18.1	23.72 ± 13.2/25.60 ± 17.2	
Post-pre difference	4.00 ± 0.33/2.59 ± 0.5	2.24 ± 1.2/2.04 ± 0.6	>0.05 ^K^/>0.05 ^K^
**Box and Block Score**			
(L/R)	59.88 ± 14.84/54.64 ± 22.28	65.40 ± 13.17/63.21 ± 16.22	
(count of cubes during 60 s)	63.11 ± 15.50/59.70 ± 21.61	69.20 ± 15.21/66.75 ± 13.91	
Post-pre difference	3.94 ± 0.68/5.06 ± 0.39	3.81 ± 2.11/3.55 ± 2.34	>0.05 ^K^/>0.05 ^K^

Values are represented as the median (interquartile range) or the mean ± SD. ^A^ ANOVA test. ^K^ Kruskal–Wallis test. The MAS score is presented as the number of persons having scores of 0/1/1+ points; * *p* < 0.05; NA, Not Applicable; R/L, affected Right and Left hand, respectively.

**Table 4:** Kinematic data (*n* = 42).

Variables	AG (*n* = 17)	KG (*n* = 25)	*p*-Value
Active ROM_post_–ROM_pre_			
**Shoulder**			
Flexion	19.67 ± 1.11	17.20 ± 2.32	>0.05 ^K^
Extension	6.27 ± 0.18	7.00 ± 4.45	>0.05 ^K^
Abduction	28.55 ± 4.44	5.40 ± 2.21	<0.05 ^K^*
Adduction	6.93 ± 2.77	3.60 ± 0.45	<0.05 ^K^*
Internal rotation	5.51 ± 2.03	9.6 ± 7.34	>0.05 ^K^
External rotation	5.39 ± 2.09	8.00 ± 1.71	>0.05 ^K^
**Elbow**			
Flexion	15.90 ± 6.70	10.6 ± 2.90	>0.05 ^K^
Extension	3.40 ± 5.97	0.40 ± 0.66	>0.05 ^K^
Supination	8.77 ± 1.78	1.40 ± 1.46	<0.05 ^K^*
Pronation	7.00 ± 4.02	5.80 ± 0.02	>0.05 ^K^
**Wrist**			
Flexion	6.12 ± 3.34	6.80 ± 2.16	>0.05 ^K^
Extension	7.26 ± 0.61	4.24 ± 0.55	>0.05 ^K^
Ulnar deviation	1.89 ± 0.29	4.80 ± 0.16	>0.05 ^K^
Radial deviation	0.64 ± 0.81	4.20 ± 1.11	>0.05 ^K^

ROM_pre_–ROM_post_ are clinical measures of the stroke-affected arm before and after the treatment session, respectively. ROM_pre_–ROM_post_ is the change or effect of the treatment. Values are represented as the median (interquartile range) or the mean ± SD. ^K^ Kruskal–Wallis test. * *p* < 0.05.

**Table 5:** Cognitive function data (*n* = 36).

MMSE Score	Pre-	Post-	Post-Pre Difference	*p*-Value
**Total Score**				
AG (*n* = 17)	24.05 ± 2.43	26.51 ± 2.09	4.82 ± 3.45	
KG (*n* = 19)	23.21 ± 2.27	24.36 ± 1.46	2.21 ± 2.97	<0.05 ^A^*
**Orientation (To Time, To Place)**				
AG	4.32 ± 0.36	4.56 ± 0.65	0.17 ± 0.39	
KG	4.15 ± 0.70	4.28 ± 0.72	0.15 ± 0.37	>0.05 ^K^
**Registration**				
AG	2.29 ± 0.66	2.41 ± 0.69	0.11 ± 0.33	
KG	2.10 ± 0.78	2.26 ± 0.71	0.15 ± 0.37	>0.05 ^K^
**Attention and Calculation**				
AG	3.01 ± 1.13	3.58 ± 0.91	0.57 ± 0.71	
KG	2.83 ± 1.01	3.05 ± 1.14	0.21 ± 0.67	<0.05 ^K^*
**Recall**				
AG	2.23 ± 0.81	2.52 ± 0.60	0.29 ± 0.58	0
KG	2.10 ± 0.99	2.31 ± 0.86	0.21 ± 0.63	>0.05 ^K^
**Language**				
AG	1.88 ± 0.32	1.94 ± 0.23	0.05 ± 0.42	
KG	1.84 ± 0.36	1.89 ± 0.30	0.05 ± 0.22	<0.05 ^K^*
**Repetition**				
AG	0.88 ± 0.32	1.70 ± 0.66	0.83 ± 0.72	
KG	0.89 ± 0.30	0.94 ± 0.22	0.52 ± 0.40	>0.05 ^K^
**Complex Commands**				
AG	1.25 ± 1,05	1.99 ± 0.89	0.84 ± 0.94	
KG	1.30 ± 1.94	1.32 ± 1.95	0.03 ± 0.56	<0.05 ^K^*

Values are represented as the median (interquartile range) or the mean ± SD. ^K^ Kruskal–Wallis test, AANOVA, * *p* < 0.05.

**Table 6:** HAD testing results (*n* = 36).

HAD Score	Pre	Post	Post-Pre Difference	*p*-Value
**Depression**				
AG	5.41 ± 3.12	4.94 ± 3.09	0.47 ± 0.03	>0.05 ^K^
KG	8.40 ± 4.44	8.48 ± 4.43	0.08 ± 0.01	
**Anxiety**				
AG	5.52 ± 2.37	4.11 ± 1.93	1.41 ± 0.44	<0.05 ^K^*
KG	9.16 ± 4.59	8.64 ± 4.15	0.52 ± 0.45	

Values are represented as the mean ± SD. The Kruskal–Wallis test was used for comparison. D/A, Depression/Anxiety according to the HAD score.
